# Observing Thermal Conditions of Historic Buildings through Earth Observation Data and Big Data Engine

**DOI:** 10.3390/s21134557

**Published:** 2021-07-02

**Authors:** Athos Agapiou, Vasiliki Lysandrou

**Affiliations:** 1 Department of Civil Engineering and Geomatics, Faculty of Engineering and Technology, Cyprus University of Technology, Saripolou 2-8, Limassol 3036, Cyprus; vasiliki.lysandrou@cut.ac.cy; 2 Eratosthenes Centre of Excellence, Saripolou 2-8, Limassol 3036, Cyprus

**Keywords:** built heritage, historic buildings, thermal analysis, satellite data, Landsat space program, Google Earth Engine, Cyprus

## Abstract

This study combines satellite observation, cloud platforms, and geographical information systems (GIS) to investigate at a macro-scale level of observation the thermal conditions of two historic clusters in Cyprus, namely in Limassol and Strovolos municipalities. The two case studies share different environmental and climatic conditions. The former site is coastal, the last a hinterland, and they both contain historic buildings with similar building materials and techniques. For the needs of the study, more than 140 Landsat 7 ETM+ and 8 LDCM images were processed at the Google Earth Engine big data cloud platform to investigate the thermal conditions of the two historic clusters over the period 2013–2020. The multi-temporal thermal analysis included the calibration of all images to provide land surface temperature (LST) products at a 100 m spatial resolution. Moreover, to investigate anomalies related to possible land cover changes of the area, two indices were extracted from the satellite images, the normalised difference vegetation index (NDVI) and the normalised difference build index (NDBI). Anticipated results include the macro-scale identification of multi-temporal changes, diachronic changes, the establishment of change patterns based on seasonality and location, occurring in large clusters of historic buildings.

## 1. Introduction

Earth observation sensors have been widely used in the last two decades to observe, survey, and monitor the built heritage environment [1,2,3]. The increased capabilities of space programs initiated and operated by several national agencies and the private sector facilitated research and application around the study, modelling, and predicting of various natural and anthropogenic phenomena affecting built heritage [4,5].

Since 1999, when the first high-resolution commercial satellite sensor, namely the IKONOS was set into orbit, several other satellite sensors were launched. Most new satellite sensors can capture the visible and near-infrared parts of the spectrum (approximately between 400 and 900 nanometers). Few of the new satellite sensors can capture the mid-infrared part of the spectrum (25–40 microns), while even fewer are designed to be sensitive to the thermal spectral region. The thermal spectrum is covered by Landsat data since the 80s, after the launch of Landsat 4 [6]. Currently, both Landsat 7 and 8 are active and can provide medium-resolution thermal images.

The Landsat space program is the oldest space program designed and operated for environmental purposes. Since 1972, several space Landsat sensors have been launched in space and provide valuable multispectral datasets in a systematic way and with almost global coverage. Landsat is a joint effort of the U.S. Geological Survey (USGS) and the National Aeronautics and Space Administration (NASA) [7].

Both Landsat 7 and Landsat 8 satellites orbit the Earth at an altitude of 705 km (438 miles) in a 185-kilometre (115-mile) swath, moving from north to south over the sunlit side of the Earth, in a sun-synchronous orbit. Each satellite makes a complete orbit every 99 min, approximately 14 full orbits each day, and crosses every point on Earth once every 16 days. Although each satellite has a 16-day full-Earth-coverage cycle, their orbits are offset to allow 8-day repeat coverage of any Landsat scene area on the globe. Between the two satellites, more than 1,000 scenes are added to the USGS archive on a daily basis. The extraction of land surface temperatures from Landsat images has been studied in the recent past by [8,9].

This study advances the state of the art regarding the use of thermal images specifically for the thermal monitoring of cultural heritage sites, which is normally carried out with the support of optical data [10,11,12,13]. The use of thermal bands from satellite sensors is mentioned in [14], but it refers to the detection and identification of ancient hills in Farahan, Iran and not for monitoring purposes. As mentioned by [15], the lack of high-resolution thermal images is a limiting factor for their use in cultural heritage applications, and for this reason, the use of higher-resolution datasets is preferred [16].

In this study, we aim to explore and evidence how medium resolution satellite thermal data can be used to analyse historic buildings’ thermal conditions. This effort makes part of a holistic, integrated, multi-disciplinary initiative under the PERIsCOPE (“Portal for hERItage buildingS integration into the COntemPorary built environment”, https://uperiscope.cyi.ac.cy/ (accessed on 2 July 2021)), project umbrella, aiming to bring together technological innovation and restoration of heritage buildings. The results presented here are following the preliminary outcomes of Agapiou et al. (2021) [17]. The overall project objective is to design and develop an innovative platform for the identification, classification, documentation, and renovation of heritage buildings, which can be exploited by various stakeholders and professionals of the sector. PERIsCOPE enables the exploitation of state-of-the-art techniques in the scientific fields of building information modelling (BIM), remote sensing, terrestrial and aerial 3D modelling techniques, and non-destructive onsite testing, pursued by leading research and academic institutions of Cyprus in these fields.

The paper is organised as follows: initially, the overall methodology and the datasets used are presented (Section 2). Then, the description of the case study area follows (Section 3). Results and image processing outcomes are given in Section 4, following by a discussion (Section 5) and ending with the conclusions (Section 5).

## 2. Methodology

For the needs of the PERIsCOPE project, three different analysis scales were adopted. During the macro-scale analysis, satellite-based products are used for the overall estimation of the temperature variations on a wider area, while on a semi-macro scale analysis, low attitude sensors are employed. Finally, at the micro-scale analysis, ground measurements and techniques are used to validate the individual buildings’ conditions. This study presents the results from the macro-scale analysis, for which thermal data, optical satellite images, and ready satellite products were exploited to provide multi-temporal information for the selected urban testbeds (refer to the next section).

**Figure 1:**
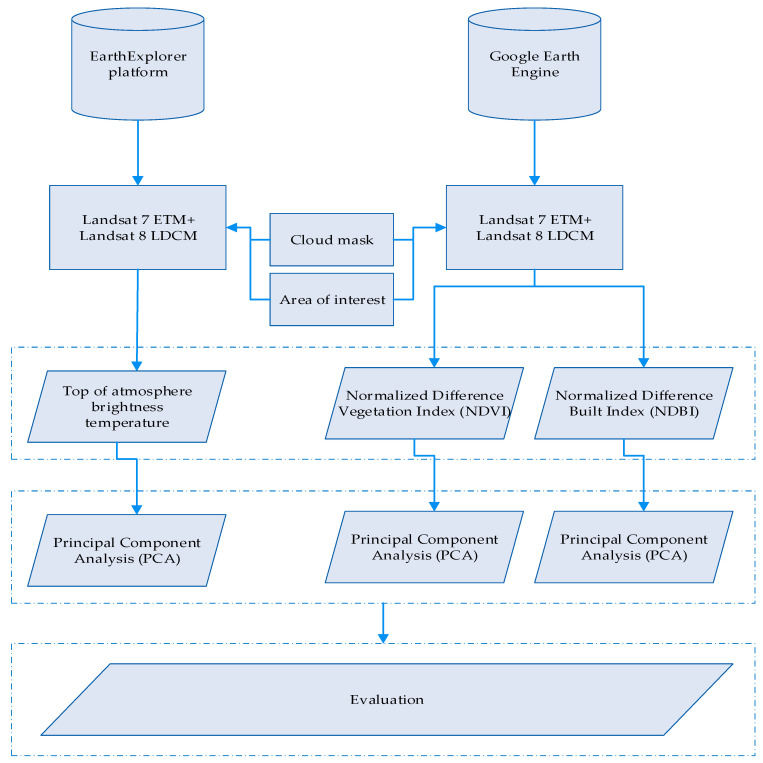
Overall methodology of the current study.

The overall methodology of this study is grouped in two parts as follows (Figure 1): the first one is related to desktop image analysis processing, while the second includes the use of big data cloud platforms. For the first part, land surface temperature (LST) estimations were extracted from Landsat archival images, while during the second, optical satellite data were processed on cloud platforms to produce various products such as the normalised difference vegetation index (NDVI) and the normalised build area index (NDBI).

### 2.1. Thermal Image Processing

Landsat 7 and 8 archives were downloaded through the EarthExplorer platform [18], found under the Landsat menu in the “Landsat Collection 1 Level-1” section, in the “Landsat 7 Enhanced Thematic Mapper Plus (ETM+) Level-1” and “Landsat 8 OLI/TIRS C1 Level-1” datasets. Newly acquired Landsat 8 scenes are available for download within 24 h of data acquisition.

The Landsat Collections Level-1 data downloaded from the EarthExplorer platform were rescaled to the top of atmosphere (TOA) reflectance and/or radiance using radiometric rescaling coefficients, provided in the metadata file that is delivered with the Level-1 product (metadata—MTL file). The metadata file also contains the thermal constants needed to convert thermal band data to TOA brightness temperature. Landsat Collections Level-1 data products consist of quantised and calibrated scaled digital numbers (DN). These numbers represent the multispectral image data. Landsat 8 products acquired data by both the operational land imager (OLI) and thermal infrared sensor (TIRS) are delivered in 16-bit unsigned integer format. Landsat 1–7 products are generated from single sensor data and are delivered in an 8-bit unsigned integer format.

More than 140 satellite images were selected (upon cloud coverage), downloaded, and processed, covering the period between 2013 and 2020. Specifically, we have used 16 images during the Winter season, 30 images over Spring, 57 images for Summer, and 38 during Autumn. These variations in terms of available thermal datasets between the seasons were expected, mainly due to the cloud coverage. The dataset includes images both from the Landsat 7 ETM+ sensor and the Landsat 8 LDCM sensor. The spectral radiance of these data’s thermal band was converted to TOA brightness temperature, using the thermal constants in the MTL file:

(1) $$T=\frac{K_{2}}{\ln\left(\frac{K_{1}}{L_{\lambda }}+1\right)}$$

where:

*T* = Top of atmosphere brightness temperature (K), where:
*Lλ* = TOA spectral radiance (Watts/(m2 × srad × μm))
*K*1 = Band-specific thermal conversion constant from the metadata (K1_CONSTANT_BAND_x, where x is the thermal band number)
*K*2 = Band-specific thermal conversion constant from the metadata (K2_CONSTANT_BAND_x, where x is the thermal band number)

From the above-described desk-based analysis, the surface temperatures of the areas of interest were achieved. The various conversions and corrections (i.e., conversion to TOA radiance, reflectance, and top of atmosphere brightness temperature) led to the development of a series of thematic maps (refer to Section 4: Discussion) in a GIS environment where spatial analysis was also implemented. Through this environment, mean temperatures and standard deviation maps were generated. In addition, the principal component analysis (PCA) was performed. PCA is a statistical analysis that takes into account the variations within the image [19]. This analysis can be applied to a multi-temporal dataset to include the temporal variance. In this study, both the NDVI and the NDBI variances are estimated. Therefore, the PCA is used as a change detection method in cases where the radiometric noise is minimal [20].

### 2.2. Optical Data Processing

For the optical data processing, the researchers used the Google Earth Engine cloud platform. The specific platform permits the use and management of hundreds of satellite data. The Google infrastructure was used to extract optical products, namely the NDVI and the NDBI indices, which characterise the vegetated and built-up areas, respectively. The equations for the two indices are presented below.

(2) $$NDVI=\left(\rho_{NIR}-\rho_{red}\right)/\left(\rho_{NIR}+\rho_{red}\right)$$

(3) $$NDBI=\left(\rho_{SWIR}-\rho_{NIR}\right)/\left(\rho_{SWIR}+\rho_{NIR}\right)$$

where, $\rho_{NIR}$ refers to the reflectance value at the near-infrared part of the spectrum, $\rho_{red}$ refers to the reflectance value at the red part of the spectrum, and $\rho_{SWIR}$ refers to the short-wave infrared reflectance value at the near-infrared part of the spectrum. Based on the obtained NDVI and NDBI indices for the areas under examination, time-series annual maps, starting from 2013 until 2020, were created. These maps were used for a diachronic interpretation and evaluation of the changes that occurred in the landscape of the two testbeds.

Once again, PCA was applied to these outcomes to showcase where significant changes occurred during the period 2013–2020. Any changes were then correlated with the results of the temperature variations obtained from the thermal analysis.

## 3. Case Study Area

**Figure 2:**
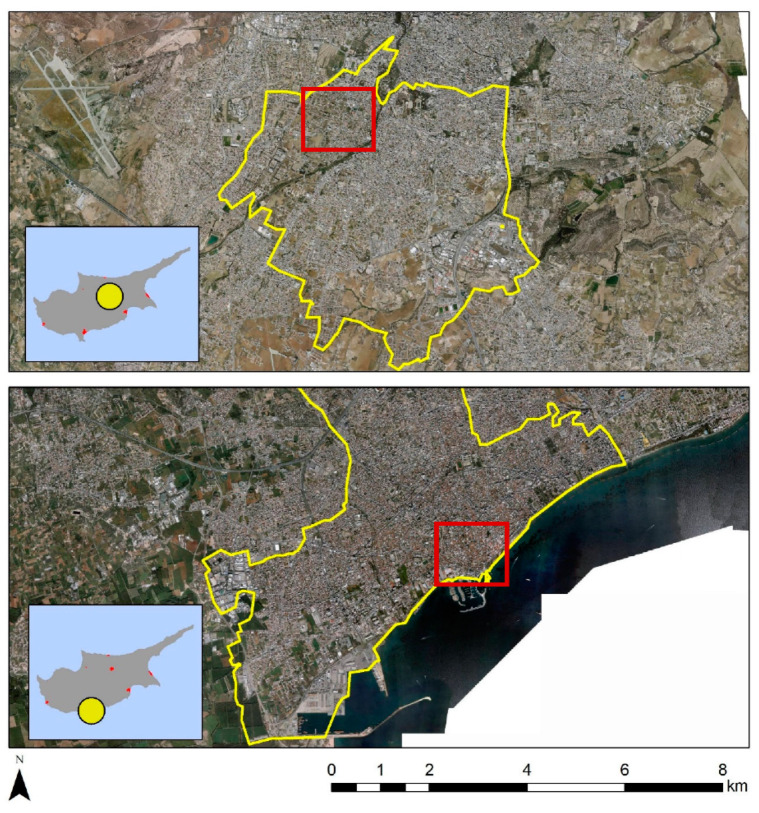
The municipalities of Strovolos (**top**) and Limassol (**bottom**) in Cyprus, selected as case studies. Red polygons include specific historic buildings under investigation here.

Two different pilot testbeds have been selected in two different districts in Cyprus, namely (a) the old Strovolos core in Nicosia District and (b) the Cami Cedid and Arnaut historic cores in Limassol District (Figure 2). The two study areas include a variability regarding the architectural typology of the historic buildings, while at the same time, they share different environmental and climatic conditions. Case study (a) is located inland, approximately in the centre of the island, while case study (b) is on the south coast. The different environment discloses the thermal variations. Together with the study of the building material properties on individual buildings (done by another team working for the Periscope project), any potential differences in how buildings behave in each environment are expected to be revealed.

**Figure 3:**
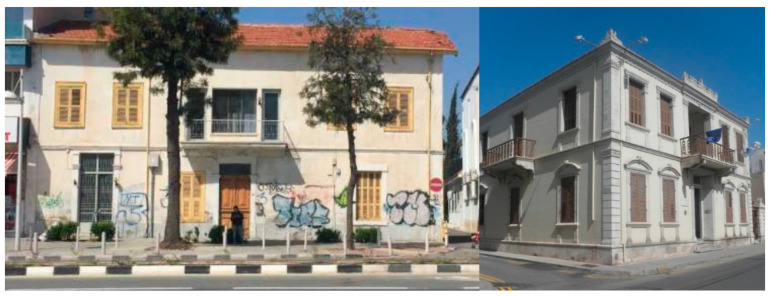
Examples of historic buildings in Limassol, investigated under the Periscope project (photograph sources: Municipality of Limassol©).

In each of these areas, ten individual buildings will be selected for further investigation. The project consortium members will apply a series of ground investigation techniques and methods, including 3-D geometrical documentation, thermal, and architectural analysis on the specific buildings. Examples from the historic buildings of Limassol are shown in Figure 3.

## 4. Results

### 4.1. Thermal Analysis

As stated above, more than 140 Landsat 7 and Landsat 8 thermal images were downloaded from the USGS Earth Explorer platform. The digital numbers of Landsat data products were converted into Kelvin units, using Equation (1) (refer to Section 2). It is underlined that from these multi-temporal datasets, it is possible to extract individual thermal images for specific dates or seasons for both areas.

**Figure 4:**
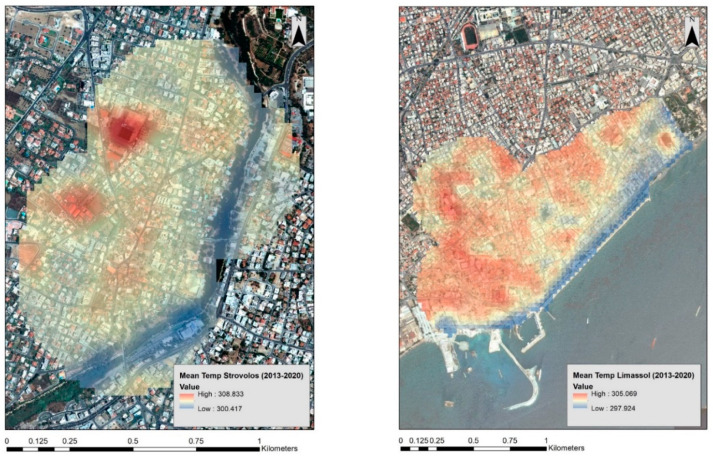
(**Left**) Mean temperature over the Strovolos area between the years 2013 and 2020. (**Right**) Mean temperature over the Limassol area between the years 2013 and 2020. The red colour indicates higher mean temperatures, while the blue colour indicates lower mean temperatures.

**Figure A1:**
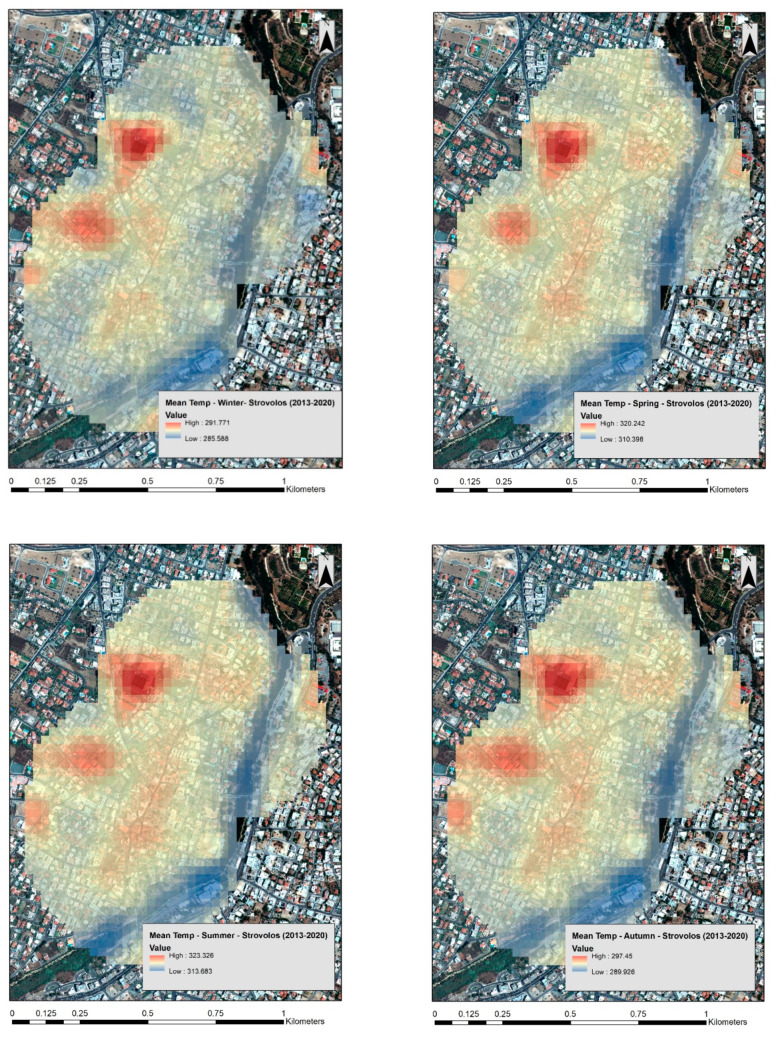
Seasonal mean temperature over the Strovolos area, between the years 2013 and 2020. The red colour indicates higher mean temperatures, while the blue colour indicates lower mean temperatures.

**Figure A2:**
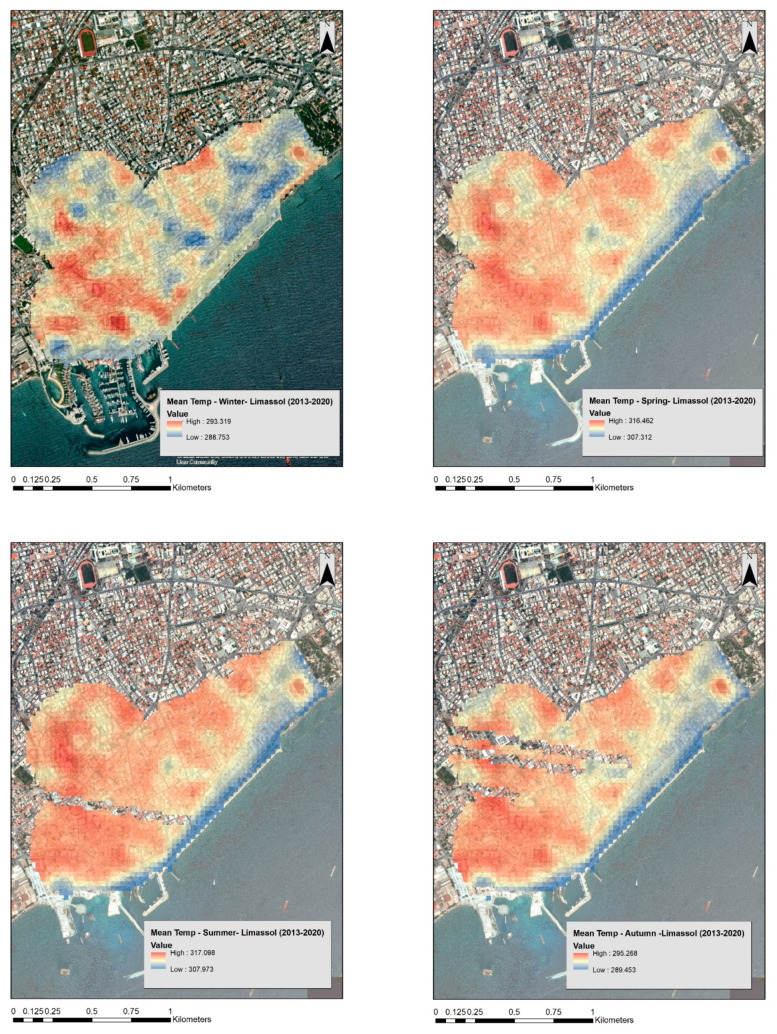
Seasonal mean temperature over the Limassol area, between the years 2013 and 2020. The red colour indicates higher mean temperatures, while the blue colour indicates lower mean temperatures.

All data were then imported into the ArcGIS environment to visualise the results and to apply spatial analysis. Figure 4 (left) shows the mean temperature results for the period 2013 to 2020, over case study (a), the Strovolos area. Higher mean temperatures are shown with red colour, and lower mean temperatures are visualised with blue. It can be observed that lower temperatures are recorded along the Pediaios river. In addition, two hot spot areas with high mean temperatures are marked in the western part, with red colour. A different pattern is observed over the Limassol case study (Figure 4, right), where high mean temperatures are recorded almost in the entire area of investigation, except for the coastline on the south. An overall observation is that the Strovolos area revealed approximately 3 Kelvin degrees higher mean temperature in comparison to the Limassol case study. Even though these estimated temperatures cannot be considered unconditionally representative due to the variations of the available images used per season (see Figure A1 and Figure A2), still a direct comparison between the two case studies can be made since the same Landsat images were observing both case studies in a single image.

**Figure 5:**
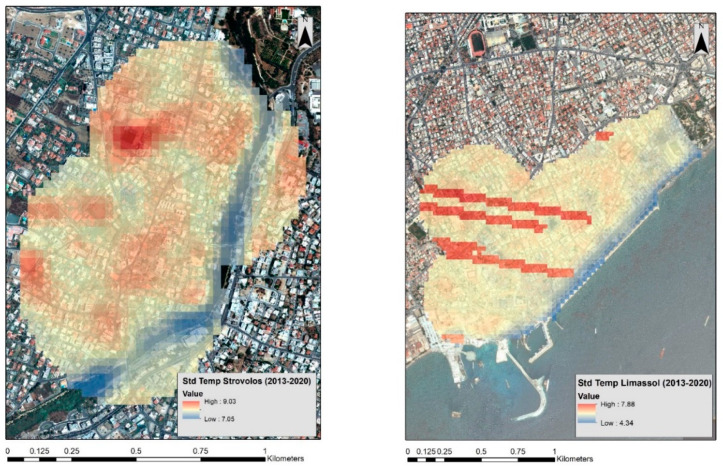
(**Left**) Standard deviation temperature over the Strovolos area between the years 2013 and 2020. (**Right**) Standard deviation temperature over the Limassol area between the years 2013 and 2020. Higher standard deviation is visualised with red colour while lower min temperatures are visualised with blue colour.

Figure 5 shows the standard deviation of the temperatures between the period 2013 to 2020 for both case studies. Standard deviation can be used to observe significant fluctuations of temperatures. Significant changes in the temperature are recorded in the Strovolos case study (Figure 5, left), following a similar pattern with the mean temperature outcomes (Figure 4, left). These fluctuations are within a range of two Kelvin degrees. In contrast, fewer fluctuations are observed in the Limassol area from 2013 to 2020 (Figure 5, right). The red lines in Figure 5 (right) are due to missing data from the Landsat 7 sensor [21].

### 4.2. Optical Data

**Figure 6:**
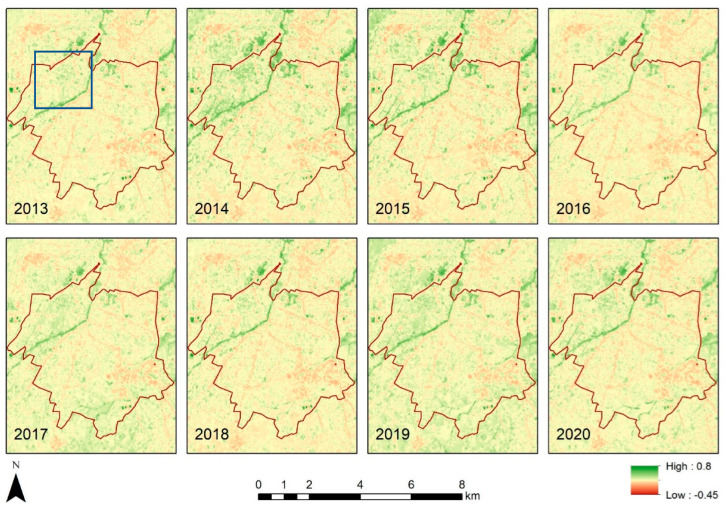
NDVI maps for the case study (a) (Strovolos) per year.

**Figure 7:**
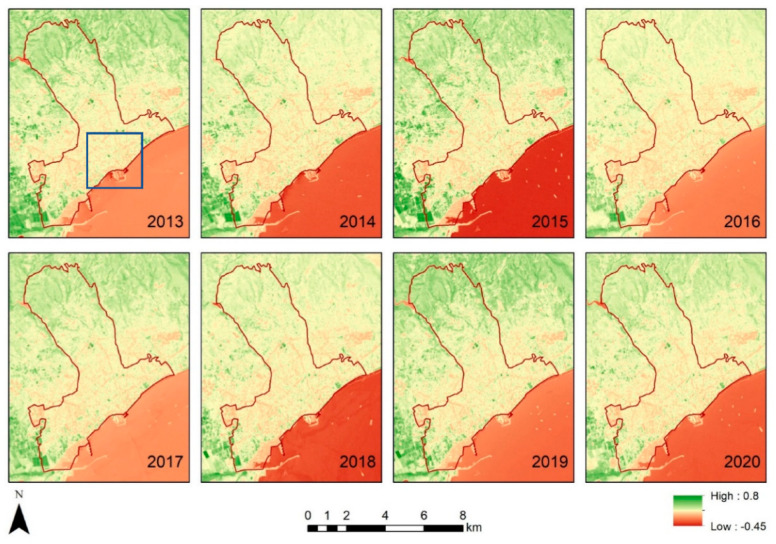
NDVI maps for Limassol case study (b) (Limassol) per year.

Using the optical bands of the Landsat sensors, the NDVI index was calculated for each year starting from 2013 to 2020. This was carried out in the Google Earth Engine platform. Figure 6 shows the results for the Strovolos case study, while Figure 7 the NDVI results of the Limassol area. The area under investigation of the previous section is shown with a blue rectangle in Figure 6 and Figure 7. Higher NDVI values close to 1 indicate areas covered with healthy vegetation, whereas areas with NDVI values, less than 0.20, indicate non-vegetated areas. Vegetated areas are highlighted with green colour compared to the rest areas shown in yellow and red.

Therefore, in Figure 6, the river basin is covered with green colour, suggesting the presence of vegetation. Differences per year can be observed within the Strovolos municipality (comparing, for instance, the years 2014 and 2020). In this pair, we get vegetation coverage on the western part of the river for the year 2014, while for the year 2020, vegetation coverage is less.

In the Limassol case study (Figure 7), differences in vegetation presence are observed in the western part of the municipality, which is primarily linked to seasonal cultivation. The differences recorded on the northern site are due to natural environmental changes (semi-mountainous area).

**Figure 8:**
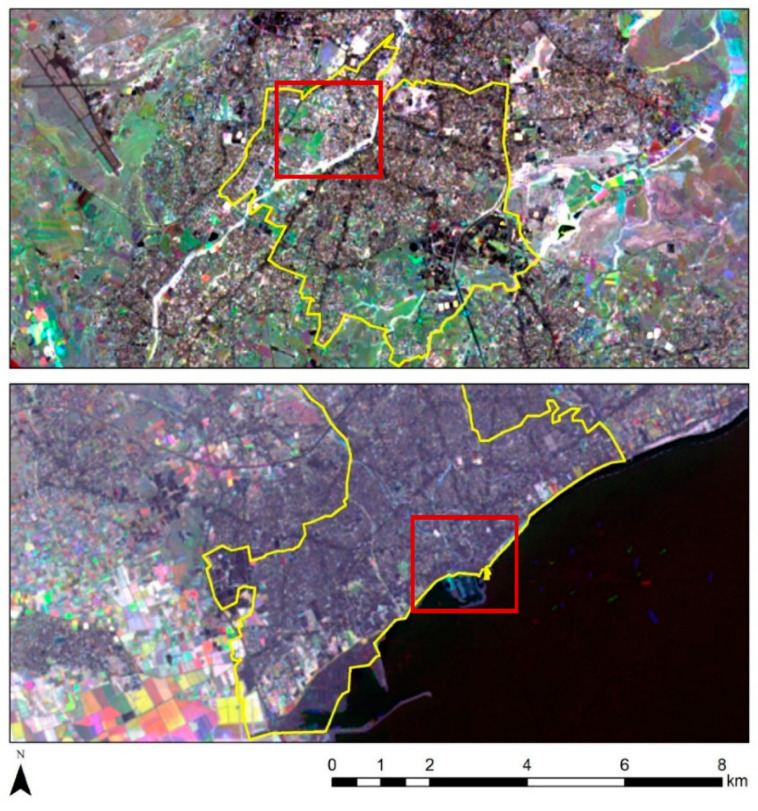
Pseudo-colour RGB composites from the NDVI annual maps, using 2013 (B), 2017 (G), and 2020 (R) as reference years. On top, the results for the Strovolos case study, and on the bottom the results from the Limassol case study.

Based on these annual NDVI maps, differences per year can be estimated. The following figure (Figure 8) reveals differences in the landscape based on RGB pseudo-colour composites from the NDVI maps for the years 2013, 2017, and 2020 for both cases studies (Strovolos and Limassol). Areas with blue colour indicate high NDVI values for the year 2013, areas with green colour show pixels with high NDVI values for the year 2017, while high NDVI values for the year 2020 are visualised in red. White tones in Figure 8 indicate areas with similar NDVI during the three years. For instance, the Pediaios river, in the Strovolos case study, is highlighted in white for all three years (Figure 8, top). The same as for some agricultural fields in the western part of Limassol (Figure 8, bottom).

For the specific areas within the red rectangle, some changes are noted between the years 2017 and 2020 in the Strovolos case study (Figure 8, top, red rectangle), while in the Limassol area, no significant changes are recorded (Figure 8, bottom, red rectangle). These changes could be a result of a land-use change or seasonal changes.

**Figure 9:**
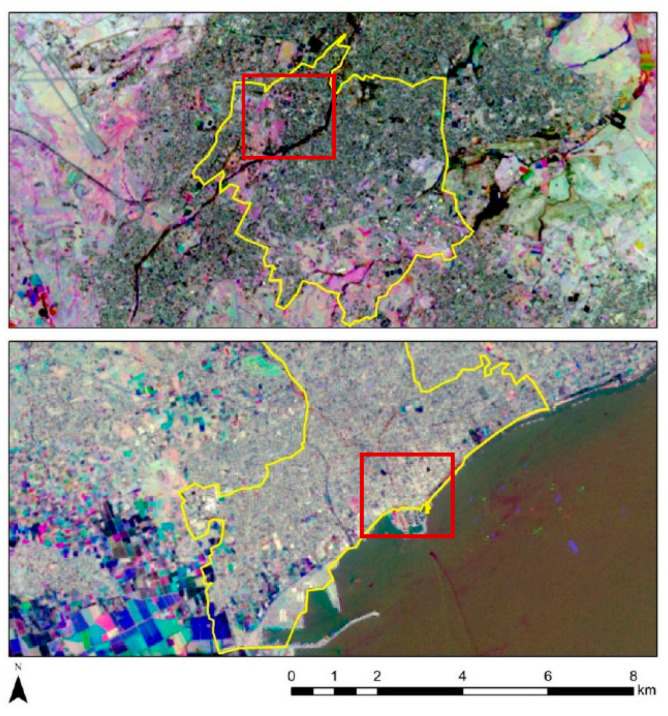
Pseudo-colour RGB composites from the NDBI annual maps using the reference years of 2013 (B), 2017 (G), and 2020 (R). On top is the results from the Strovolos case study and on the bottom the results from the Limassol case study.

The NDBI index was calculated per year, following Equation (3) (refer to Section 2, above), for both Strovolos and Limassol case studies, using the short-wave infrared and the near-infrared part of the spectrum. Similar results as those of the NDVI index (Figure 6 and Figure 7) have been generated for each year. For detecting changes throughout this period, a pseudo-colour composite was created (Figure 9). As before, high NDBI values for the year 2013 are shown with blue colour, high NDBI values for the year 2017 are shown with green colour, while high NDBI values for the year 2020 are shown in the red band (see purple in Figure 9). Land-use change has been recorded based on the NDBI index for the year 2020 (red rectangle, Figure 9, top). These changes match the observations made regarding the mean temperatures of the area (see high mean temperatures in Figure 4).

**Figure 10:**
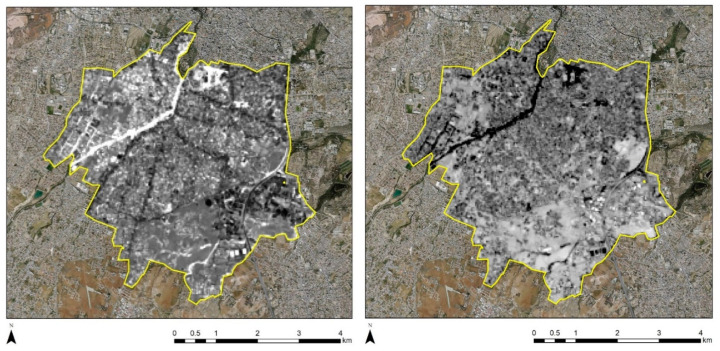
PCA analysis for the NDVI (**left**) and the NDBI (**right**) over the Strovolos case study.

In order to further evaluate the temporal changes, the PCA was applied for the NDVI and the NDBI indices for the Strovolos and Limassol case studies. Figure 10 displays the results of the first principal component (PC1) for the NDVI (left) and the NDBI (right) indices. Higher PC1 values are indicated with a white tone of grey in Figure 10, while lower PC1 values are indicated with a black tone of grey. Therefore, pixels with bright tones of grey indicate the presence of significant changes in the NDVI and the NDBI index during the period 2013 until 2020. As we see in Figure 10 left, changes recorded from the NDVI values are along with the river stream and to its north-western part (the area that includes the historic buildings under examination). The seasonal vegetation variations are expected and are connected to the river. Regarding the NDBI index (Figure 10, right), changes were recorded in the north/east area, at a fair distance from the historic core of Strovolos. Therefore, these changes are probably related to modern building development.

**Figure 11:**
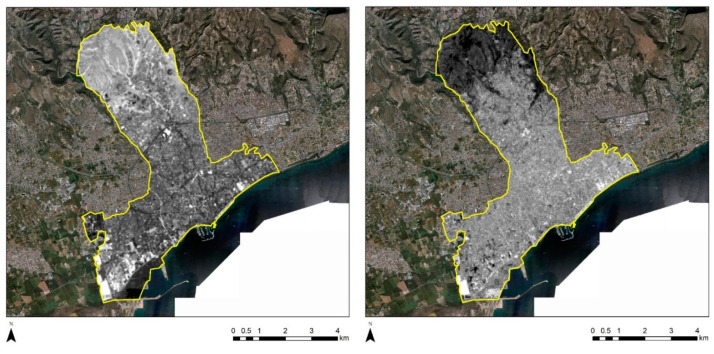
PCA analysis for the NDVI (**left**) and the NDBI (**right**) over the Limassol case study.

A similar approach was also followed for the Limassol case study. The results are shown in Figure 11 for the NDVI (left) and NDBI (right) indices. In this area, we can observe changes along the coastline. These are highlighted with white tones of grey. These changes should be linked with new constructions carried out in the last years at the seafront of the city, where sky-towers were constructed [22].

## 5. Discussion

The previous section presented the results from the thermal analysis of more than 140 Landsat images. In addition, the outcomes from the processing of two indices using the optical spectral bands of the sensor, namely the NDVI and the NDBI indices, were also presented.

The processing of the thermal analysis indicated some hot spot areas in the case study of Strovolos. In contrast, in Limassol, several areas were detected with high mean temperature. It should be noted that a difference of approximately 3 degrees Kelvin has been observed between the Strovolos and Limassol case studies, with the higher temperature values recorded in the first case study, as expected, since Strovolos is in the hinterland. Of course, the temperature differences are also dictated by the season (see Figure A1 and Figure A2).

The space-based observation allowed the detection of multitemporal changes for eight years, starting from 2013 to 2020. Despite Landsat medium spatial resolution (100 m), the benefits of using space-based observations are evident since they supported the analysis in the broader context of both case studies.

A similar approach was also implemented through the Google Earth Engine big data cloud platform, where Landsat data were processed in order to extract the NDVI and the NDBI indices. The results from this analysis evidenced that the NDBI index was sensitive, and therefore able to capture the thermal variations of the Strovolos case study. Indeed, through the PCA analysis, significant changes during the period 2013 to 2020 were recorded, fully in line with the hot spot thermal areas of the Strovolos case study.

Hereunder, an example is displayed of how the above-described research could be employed to support local models for estimating thermal conditions of historic clusters. The Strovolos area is used as a case study.

**Figure 12:**
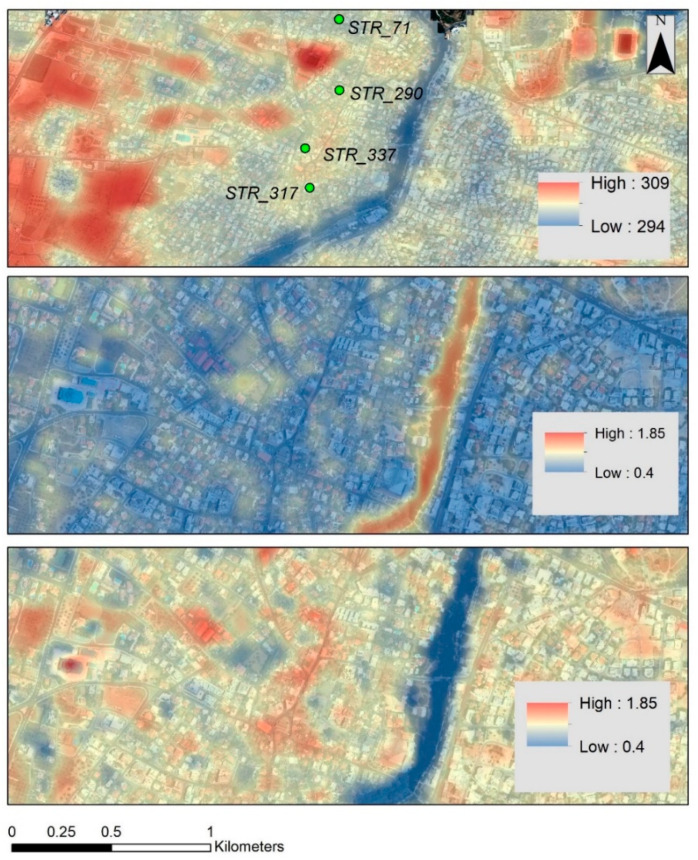
(**Top**) Mean thermal temperature over the Strovolos case study. (**Middle**) The PC1 analysis of the NDVI of the same area. (**Bottom**) The PC1 analysis of the NDBI of the same area (period 2013–2020). Selected historic buildings are shown on top (STR_XX).

Figure 12 (bottom) shows that the NDBI index has a good correlation with the area’s thermal response. Indeed, the red hotspots, which are visible in Figure 12 bottom, and which equal augmented building activity, are matching the red hotspots of Figure 12 top, which are the result of recorded high temperatures. The NDVI index tends to provide a “reverse” outcome. This is very important, as, in many satellite sensors, the thermal spectral band is missing. In contrast, the short-wave infrared, near-infrared and red spectral bands used for the calculation of the NDBI and NDVI are more frequently found in satellite sensors, even with higher spatial resolution.

**Figure 13:**
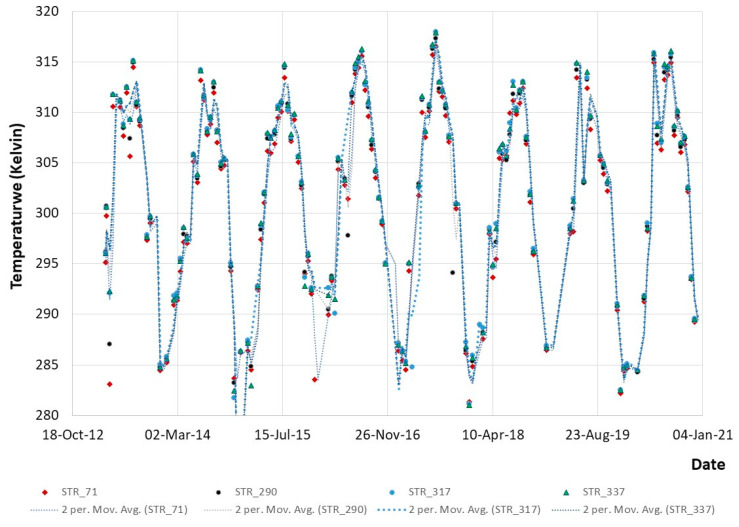
Temperature profile (in Kelvin) for four historic buildings: STR_71; STR_290; STR_317; and STR_337 (see Figure 12), as reported from the Google Earth Engine for the period 2013–2020.

In addition, satellites facilitated the extraction of individual temperatures for specific historic buildings (within the areas of interest), as indicated in Figure 13 below. The figure shows the temperature of four selected buildings (marked in Figure 12 as STR_71, STR_290, STR_337, and STR_317) for the period of 2013 until 2020. Even though a similar pattern is observed for all buildings, some subtle differences between them are noted. Recorded temperatures range between 285 and 315 Kelvin degrees. As expected, increased temperatures are recorded during the summer season, while they decrease slightly moving into the winter season.

**Figure 14:**
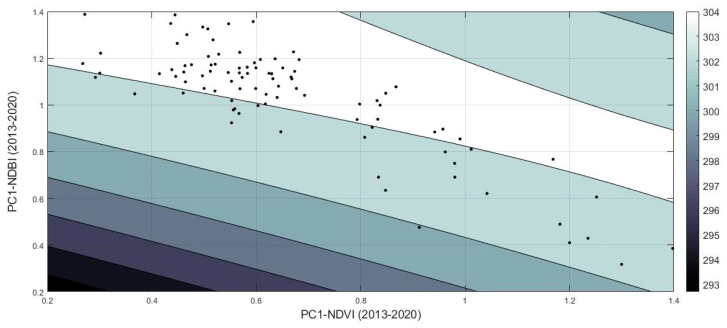
Second-order polynomial model for the estimation of the mean temperature over the Strovolos case study, using the first principal component of the NDVI in the *X*-axis and the first principal component of the NDBI in the *Y*-axis.

An empirical second-order polynomial equation was carried out for the given area (Figure 14). It was formulated using three input parameters: the mean temperature and the first principal component analysis (PC1) of the NDVI and the NDBI indices. The R-square was estimated to be 0.83, and the RMSE was found as 0.45. In detail, the coefficient of fitness was calculated as follow: SSE: 19.62; R-square: 0.8307; RMSE: 0.4568. The model is given in Equation (4) below:

Mean temperature= p00 + p10 × x + p01 × y + p20 × x^2^ + p11 × x × y + p02 × y^2^ (4)

where x (PC1 of the NDVI for the years 2013–2020) is normalised by mean 0.6655 and std 0.2407, and where y (PC1 of the NDBI for the years 2013–2020) is normalised by mean 1.03 and std 0.2347. The coefficients, with 95% confidence bounds, are as follow: p00 = 304.2, p10 = 0.2457, p01 = 0.6405, p20 = −0.142, p11 = −0.5146, and p02 = −0.3724.

The space-based observation carried out allows a first understanding of the environmental context of the historic buildings. It has the benefit of recording phenomena in large areas and providing information through time. For the interpretation of the various changes, a blending with low altitude sensors (which monitor smaller areas), as well as with ground-based recordings for an individual building, is considered an asset. The latest can also be used as ground truth validation results on occasion.

## 6. Conclusions

This paper is a follow-up research work presented in [17] under the PERIsCOPE project. The research employed satellite-based images for detecting hot spot areas regarding the thermal conditions of historic buildings in Cyprus. Regarding this, both the thermal band and the red, near-infrared and the short-wave infrared part of the spectrum from the Landsat sensors products were used. This study is relevant for the preservation and study of historic buildings in terms of contributing to the creation of a “Portal for heritage buildings integration into the contemporary built environment”, by exploiting state-of-the-art techniques for data acquisition and analysis, related to the buildings per se and their environment.

Thermal maps over two case study areas in Cyprus have been produced covering a period from 2013 until 2020. The mean temperature was estimated from this dataset that includes more than 140 thermal images. This analysis was able to detect hotspot areas that tend to give higher mean temperatures. In addition, thermal differences were observed between the two different case studies, and a primary interpretation was given.

Moreover, the NDVI and the NDBI indices were estimated and compared with the previous results. These time-series analyses allowed for a more detailed temporal mapping of the changes, while the PCA analysis highlighted areas that have significantly changed in the recent past. The NDBI index showed a good correlation with the mean thermal temperatures.

The thermal conditions of historic buildings, and specifically the seasonal thermal variations, are related to conservation needs, with possible hazardous effects in the case of thermal leaps throughout a single day. Sudden and intense variations of temperature in a small period provoke thermal shock to the materials, occasionally resulting in their cracking/fragmentation. Therefore, systematic recording of temperatures and other climatic conditions (i.e., relative humidity) in the direct environment of archaeological sites and/or historic buildings, together with measurements related directly to the construction materials and techniques, could support the planning of future preservation or restoration interventions, accordingly.

The overall outcomes of this study will be integrated with the ground investigation and other measurements on individual historic buildings to estimate a comprehensive thermal and general condition, as mandated by the PERIsCOPE project. More specifically, future research will include the analysis for seasonal changes in more detail (for an indicative seasonal heat change pattern, refer to Figure A1 and Figure A2 in Appendix A) using thermal observations from space and the correlation between building techniques, material, and thermal conditions of the buildings will be searched.

## Data Availability

Landsat data used in this study can be accessed through the EarthExplorer platform, https://earthexplorer.usgs.gov/ (accessed on 2 July 2021). The data were processed through the oogle Earth Engine big data cloud platform, https://earthengine.google.com/platform/ (accessed on 2 July 2021).
